# Multiregional neural pathway: from movement planning to initiation

**DOI:** 10.1038/s41392-022-01021-y

**Published:** 2022-06-08

**Authors:** Xing-feng Mao, Shuai-shuai Wang, Feng Han

**Affiliations:** 1 Key Laboratory of Cardiovascular & Cerebrovascular Medicine, Drug Target and Drug Discovery Center, School of Pharmacy, Nanjing Medical University, Nanjing, China; 2 Gusu School, Nanjing Medical University, Suzhou Municipal Hospital, The Affiliated Suzhou Hospital of Nanjing Medical University, Suzhou, China; 3 Institute of Brain Science, The Affiliated Brain Hospital of Nanjing Medical University, Nanjing, China

**Keywords:** Neuroscience, Computational biology and bioinformatics

A recent study by Inagaki et al. provides new evidence that the input from the pedunculopontine nucleus/midbrain reticular nucleus (PPN/MRN) to the thalamus can trigger a rapid switch of ALM activity from a motor planning mode to a motor execution mode.^1^

**Fig. 1:**
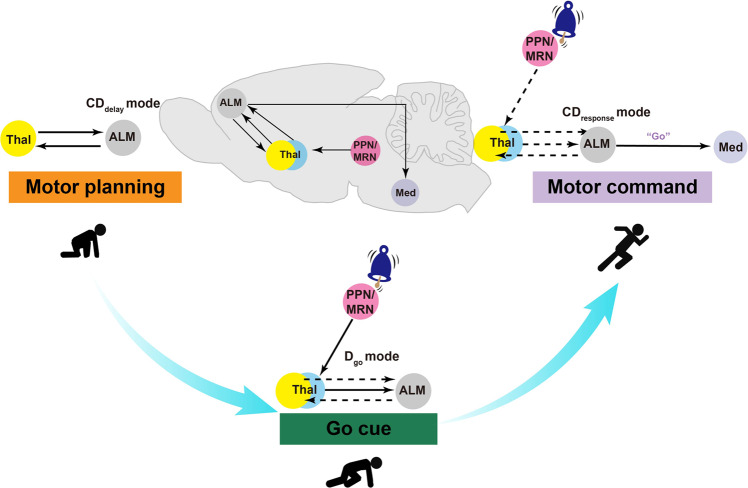
Multiregional neural pathway underlying cue-triggered movement initiation. Motor planning is maintained in a thalamus-ALM circuit. The PPN/MRN receives the “Go cue” and then activates neurons in the thal_ALM_. The Go cue triggers a rapid transition from motor planning to motor command, which activates the ALM-medulla circuit to initiate planned movements. Bottom dotted lines represent the circuit involved in motor planning. Right dotted lines indicate the circuits involved in motor planning and the Go cue signal

Planning and execution are vital components of motor behavior that are produced by distinct patterns of neuronal activity. Neuroscience researchers have devoted substantial effort to exploring how the precise neural pathway mediates cue-triggered population activity pattern switching for the execution of planned movements. The different patterns of neuronal population activity in the motor cortex, thalamus, brainstem, and spinal cord are related to motor planning and execution.^2,3^ However, to understand how neuronal dynamics in the cortex trigger motor execution, it is essential to explore the mechanism underlying the transition between those different modes in response to contextual Go cues (Fig. 1).

Neurons in the anterior lateral motor cortex (ALM) show persistent activity that instructs future actions.^3^ The “preparatory activity” depends on reciprocal interactions between the ALM and thalamus. The latter, in turn, acts as a key hub transmitting subcortical signals to the ALM during motor preparation. Nonetheless, the mechanism by which the activity mode of multiregional circuit coordination switches from the planning mode to the execution mode remains unclear.

The authors find that a midbrain–thalamus–motor cortex circuit signals a contextual cue to reorganize ALM dynamics and execute planned movement. Supporting this notion, Inagaki et al. showed that phasic activation of PPN/MRN neurons using optogenetics mimics the effect of the Go cue and triggers directional licking, while optogenetic inhibition of PPN/MRN neurons blocks Go cue-triggered licking. These findings of Inagaki et al. are consistent with previous studies in rats, cats and monkeys showing that lesions or silencing of the PPN/MRN prevents the initiation of cue-triggered motor responses.

This research subtly combines computational neuroscience with the dissection of “sensory-motor” neural circuits. Decoding neural circuits can guide us to explore how the nervous system calculates optimal behavioral strategies from variable and complex external stimuli.^4^ More importantly, state space analysis of neural activity can help us go beyond the notion of activation, which also provides a functional interpretation of population activity present in a specific brain region in a task.^1^

In this issue, Inagaki et al. demonstrated three different neuronal activity modes for motor planning and movement initiation by using neural electrophysiology recording in vivo combined with dimensionality reduction methods.^1^ Notably, a classification framework was used to describe the neuronal activity mode. “Go cue direction” (**D**_go_) represents the cue response, “delay coding direction” (**CD**_delay_) is a neuronal correlate of motor planning, and “response coding direction” (**CD**_response_) is a neuronal correlate of motor command. The power of the classification framework of these modes is in the ability to precisely analyse the population activity pattern during the transformation from planning to execution in response to Go cue signals and decode the process. During the delay period, the authors showed that motor-selective preparatory activities were mainly contained in CD_delay_ patterns in the activity space. The selective preparatory activities were rapidly reorganized into nonselective D_go_ patterns and direction-selective CD_response_ patterns after the onset of the Go cue. The causal relationships between different activity patterns were also confirmed by optogenetic manipulation.

These are longstanding but important questions about differences between animal manipulation and human therapeutic strategies. Indeed, freezing of gait (FOG), characterized by self-initiated motor deficits, is one of the main motor symptoms in patients with Parkinson’s disease (PD).^5^ The severity of FOG can be alleviated by external cues that initiate movement. Inagaki et al. suggested that the information from an external cue may bypass the dysfunctional basal ganglia in PD and reach the ALM through the midbrain-thalamus-cortical circuit to initiate movements, which may explain why cue-triggered movement disturbances rarely occur in PD patients.^1^ Deep brain stimulation (DBS) targeting specific neuronal populations of the PPN has become a common treatment for FOG in PD.^5^ Taking into account the “switch” role of the PPN in the execution of movement, further elucidation of the working mechanism of DBS will help to develop more effective therapeutic regimens for FOG in PD.

Motor control based on motion intention decoding has been a very important direction in the field of brain-computer interfaces and is of great significance in both theoretical research and practical applications.^4^ The approaches used in the decoding model in this study might help us to assemble the pieces of the puzzle of brain computation and to better integrate these separate disciplines for neuronal population activity dynamics analysis in various contexts.^1^ Thus, the decoding method developed by Inagaki et al. may help us not only understand the causal relations between neural activity and motor control but also improve the brain-computer interface.
